# A novel immunogenic cell death–related subtype classification and risk signature for predicting prognosis and immunotherapy efficacy in gastric cancer

**DOI:** 10.3389/fimmu.2023.1162876

**Published:** 2023-05-05

**Authors:** Bingqi Dong, Yingchao Wu, Junling Zhang, Yanlun Gu, Ran Xie, Xu He, Xiaocong Pang, Xin Wang, Yimin Cui

**Affiliations:** ^1^ Department of General Surgery, Peking University First Hospital, Beijing, China; ^2^ Department of Pharmacy, Peking University First Hospital, Beijing, China; ^3^ Institute of Clinical Pharmacology, Peking University, Beijing, China

**Keywords:** immunogenic cell death, prognosis, tumor microenvironment, immunotherapy, gastric cancer

## Abstract

The majority of gastric cancer (GC) patients are in a progressive stage at the initial stage of treatment, and the overall response rate to immunotherapy remains unsatisfactory largely due to the lack of effective prognostic biomarkers. Immunogenic cell death (ICD) was identified as a new form of regulated cell death that can activate adaptive immune responses and further promote immunotherapy efficacy. Therefore, we attempted to characterize the ICD-associated signature to stratify patients who could benefit from immunotherapy. In our study, two subgroups of patients were identified based on the data of 34 ICD-related genes extracted from The Cancer Genome Atlas database via consensus clustering. The estimated scores, stromal scores, immune scores, tumor purity, and survival rate showed significant differences between the low and high ICD groups. Then, we constructed an ICD-related risk signature, including IFNB1, IL6, LY96, and NT5E, using least absolute shrinkage and selection operator Cox regression analysis; then, high- and low-risk groups could be clearly distinguished. Notably, the risk score is a reliable predictor of the prognosis and immunotherapy outcome in GC, which was further validated in an immunohistochemistry assay. These results suggest that ICD is closely associated with the prognosis and tumor immune microenvironment in GC. Taken together, this study first constructed and validated a prognostic ICD-related signature to predict the survival and effect of immunotherapy in GC, which provided new insight for potent individualized immunotherapy strategies.

## 1 Introduction

Gastric cancer (GC) is a common malignant tumor and the leading cause of death from malignant tumors worldwide. In recent years, with the development of medical technology and improved treatment for GC, the diagnosis rate, survival time, and quality of life of early GC have all improved significantly. However, two-thirds of patients are already in a progressive stage at the time of initial treatment, and the 5-year survival rate is still less than 30%, even when treated with a combination of mainly surgical treatments (1). Therefore, to improve the survival rate of GC, more effective tumor molecular subtypes and prognostic markers need to be developed. The current status of treatment for patients with advanced GC is not optimistic, the efficacy of chemotherapy has reached a bottleneck, and different degrees of resistance (primary and acquired) are encountered during targeted therapy, which remain a major obstacle to clinical targeted therapy (2), resulting in the current treatment options for GC being quite limited. With the accumulation of evidence based on immunotherapy in advanced GC, the Chinese Society of Clinical Oncology and the European Society for Medical Oncology have adopted immunotherapy as the recommended third-line treatment for advanced GC (3). Immunotherapy can provide some survival benefits for some patients and maintain a sustained treatment effect for a long period. However, the overall objective response rate with immunotherapy is low, the efficiency of third-line immunotherapy is not high, and patients have short progression-free survival (4). It is therefore particularly important to increase the effectiveness and the proportion of patients who benefit from immunotherapy.

Immune checkpoint inhibitor (ICI) therapy is primarily responsible for enhancing antitumor immunity by targeting regulatory pathways on T cells (5). The ICI-mediated antitumor response depends on the degree of infiltration of T cells capable of recognizing and killing tumor cells. A lack of T cells in the tumor may lead to resistance to immunotherapy, and the immunosuppressive tumor microenvironment (TME) prevents the immune efficacy of programmed cell death protein 1 (PD-1) checkpoint inhibitors (6). Thus, low immunogenicity remains a major challenge for ICI therapy.

As a regulated form of cell death, immunogenic cell death (ICD) activates adaptive immune responses in immunologically active individuals (7). ICD is characterized by the release or exposure of damage-associated molecular patterns from dead tumor cells, thereby stimulating an antitumor immune response (8). Extracellularly released high mobility group box 1 (HMGB1) and ATP engage and activate antigen-presenting cells, contributing to the infiltration of tumor-specific T cells, while calmodulin calreticulin (CRT) exposed on the surface of dead cells provides an ‘eat me’ signal (9). A number of chemotherapeutic agents are known to induce ICD in cancer cells, including oxaliplatin, mitoxantrone, and adriamycin, thereby initiating an antitumor immune response and enhancing the efficacy of ICI therapy (10). Therefore, different ICD statuses can influence the efficacy of immunotherapy and may be a good marker for immunotherapy.

Herein, we pinpointed the importance of ICD genes in the TME and first identified an ICD-related risk signature to predict overall survival (OS) and immunotherapy response in GC, which showed good predictive accuracy. Collectively, this study provided potential prognostic biomarkers for GC and laid the foundation for new therapeutic targets.

## 2 Materials and methods

### 2.1 Data collection

Clinical data, transcriptional data, and mutation data were obtained from The Cancer Genome Atlas database (TCGA, https://portal.gdc.cancer.gov/) containing data from 375 stomach adenocarcinoma (STAD) patients and 32 normal samples. The STAD dataset GSE28541 was obtained from the Gene Expression Omnibus (GEO) database (https://www.ncbi.nlm.nih.gov/geo/).

### 2.2 Identification and integration of immunogenic cell death–related genes

It has been found that 34 genes are involved in the development of ICD, as described in previous reports (11). A protein−protein interaction (PPI) network of 34 ICD genes was created by STRING (https://string-db.org/), and the interaction score was set to 0.9. A differentially expressed gene (DEG) analysis of the STAD cohort was conducted with the R package “limma” (12) between ICD high and ICD low in the STAD cohort, using |log fold change (FC)| > 0.5 and adjusted p-values < 0.05 as cutoff criteria.

### 2.3 Functional enrichment analysis

After identifying DEGs between the ICD high and ICD low groups in the STAD cohort, to investigate the potential functions and signaling pathways of the DEGs, we performed Gene Ontology (GO, http://www.geneontology.org/), Kyoto Encyclopedia of Genes (KEGG, http://www.genome.jp/kegg/), and Gene Set Enrichment Analysis (GSEA, http://www.gsea-msigdb.org/gsea/index.jsp). Enrichment analysis was performed by using the R package “clusterProfiler” (13).

### 2.4 Consensus clustering

To identify molecular subtypes related to ICD, we used the R package “ConcensusClusterPlus” for consensus clustering, and the best results were obtained by evaluating the number of clusters between k = 2 and 10.

### 2.5 Mutation landscape analysis

We downloaded STAD somatic mutation data from the TCGA database and then used the R package “Maftools” to aggregate and visualize the STAD mutation landscape.

### 2.6 Survival analysis

According to clustering typing, the samples were divided into ICD high and ICD low groups, and the difference in OS between the two groups was estimated by the Kaplan−Meier method using the R packages “survminer” and “survival.” Survival curves were compared using the log-rank test. The significance threshold was defined as *p*< 0.05.

### 2.7 Construction and validation of the immunogenic cell death–related risk signature

In this study, the STAD data from the TCGA and GEO databases were used as the training set to generate the signatures, and univariate Cox regression analysis was performed to identify hub ICD genes significantly associated with patient outcomes. Then, the least absolute shrinkage and selection operator (LASSO) regression model was used to remove redundant factors and find the most significant ICD genes associated with survival based on the most appropriate λ values for the genes and their coefficients. The risk score was obtained using the following formula:


$$Risk\;score=\sum_{1}^{n}K_{n}*A_{n}$$

An is the expression level of ICD-related genes, Kn is the regression coefficient of prognosis-related genes, and n is the number of ICD-related genes.

### 2.8 Tumor immune correlation analysis

The cell-type identification by estimating relative subsets of RNA transcripts (CIBERSORT) algorithm was used to estimate the proportion of 22 different immune cell types, and furthermore, we calculated the difference in the estimate score, immune score, stromal score, and tumor purity between high- and low-risk groups based on the “bestimate” R package. The risk score and immune cell correlations were determined by Spearman’s correlation test based on RNAseq data from STAD and corresponding clinical information. Finally, the differences in immune checkpoint and HLA gene expression were calculated, and potential immunotherapeutic responses were predicted using the tumor immune dysfunction and exclusion (TIDE) algorithm.

### 2.9 Immunohistochemistry

Specimens were dewaxed in xylene, hydrated by immersion in graded alcohol, and then incubated at room temperature for 10 min with 3% hydrogen peroxide in the dark. Specimens were incubated with goat serum for 10 min after antigen repair and incubated overnight using primary anti-IFNB1 (1:400 dilution, 27506-1-AP), anti-IL6 (1:400 dilution, 21865-1-AP), anti-LY96 (1:400 dilution, 11784-1-AP), and anti-NT5E (1:400 dilution, 12231-1-AP). Samples were incubated with horseradish peroxidase (HRP)-conjugated secondary antibody for 1 h at room temperature. Then, diaminobenzidine (DAB) staining was used, and hematoxylin restaining was performed. The specimens were finally observed using a light microscope (DX45, Olympus Microsystems Ltd., Japan), and the sections were imaged at ×10 magnification. All antibodies were purchased from Proteintech (Rosemont, IL, USA). The study was approved by the Biomedical Research Ethics Committee of Peking University First Hospital (license number: 2021R054).

### 2.10 Statistical analysis

Bioinformatics analysis was carried out using R software (version 4.2.0). The analysis of data from the biology experiments section was performed using GraphPad Prism 8. Means of normally distributed variables were compared between two groups using unpaired t tests. Non-normally distributed data were compared using the Wilcoxon test. *P< 0.05, **P< 0.01, and ***P< 0.001 were considered significant.

## 3 Results

### 3.1 Expression analysis and consensus clustering of immunogenic cell death–related genes

**Figure 1:**
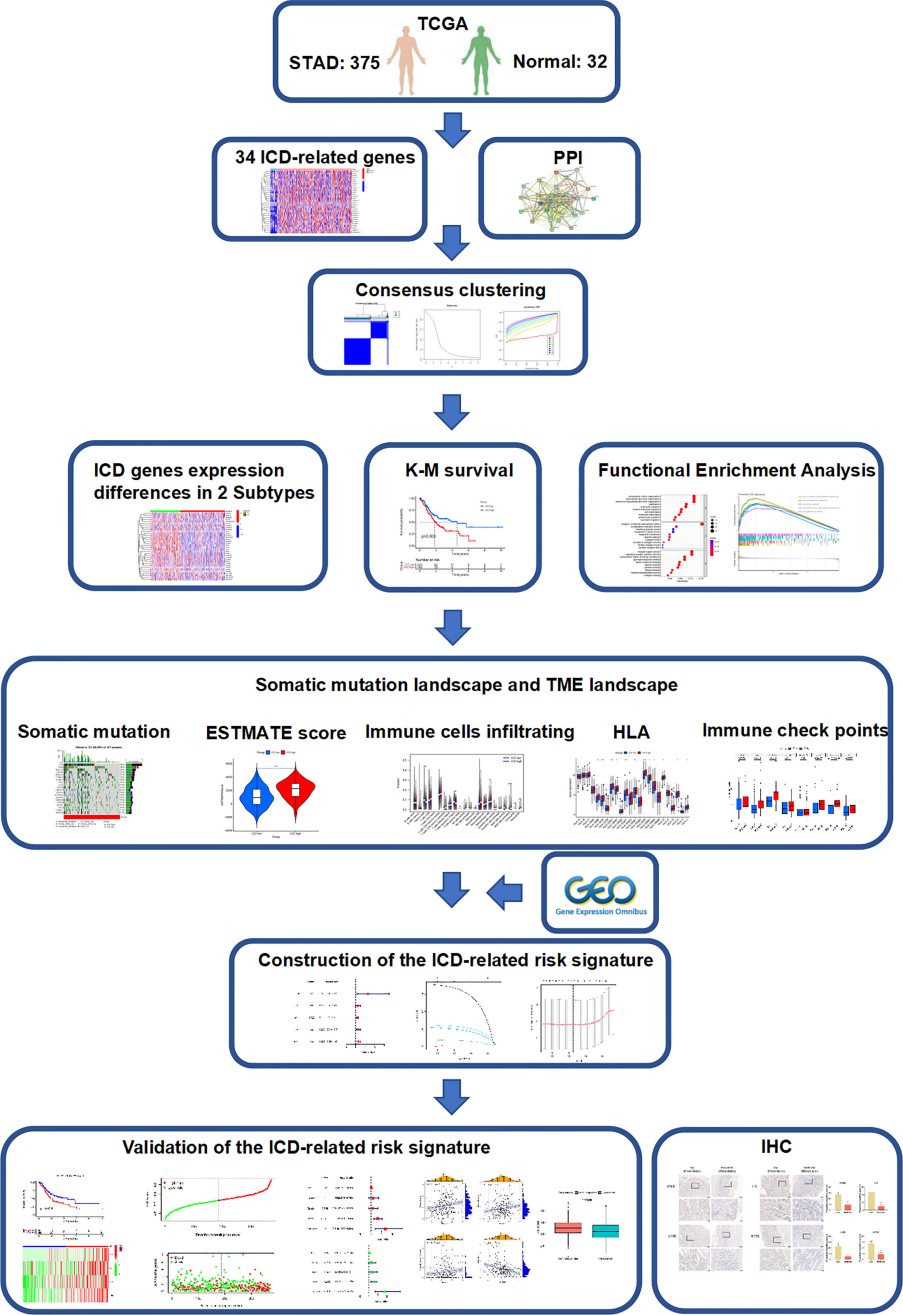
Study flow chart.

**Figure 2:**
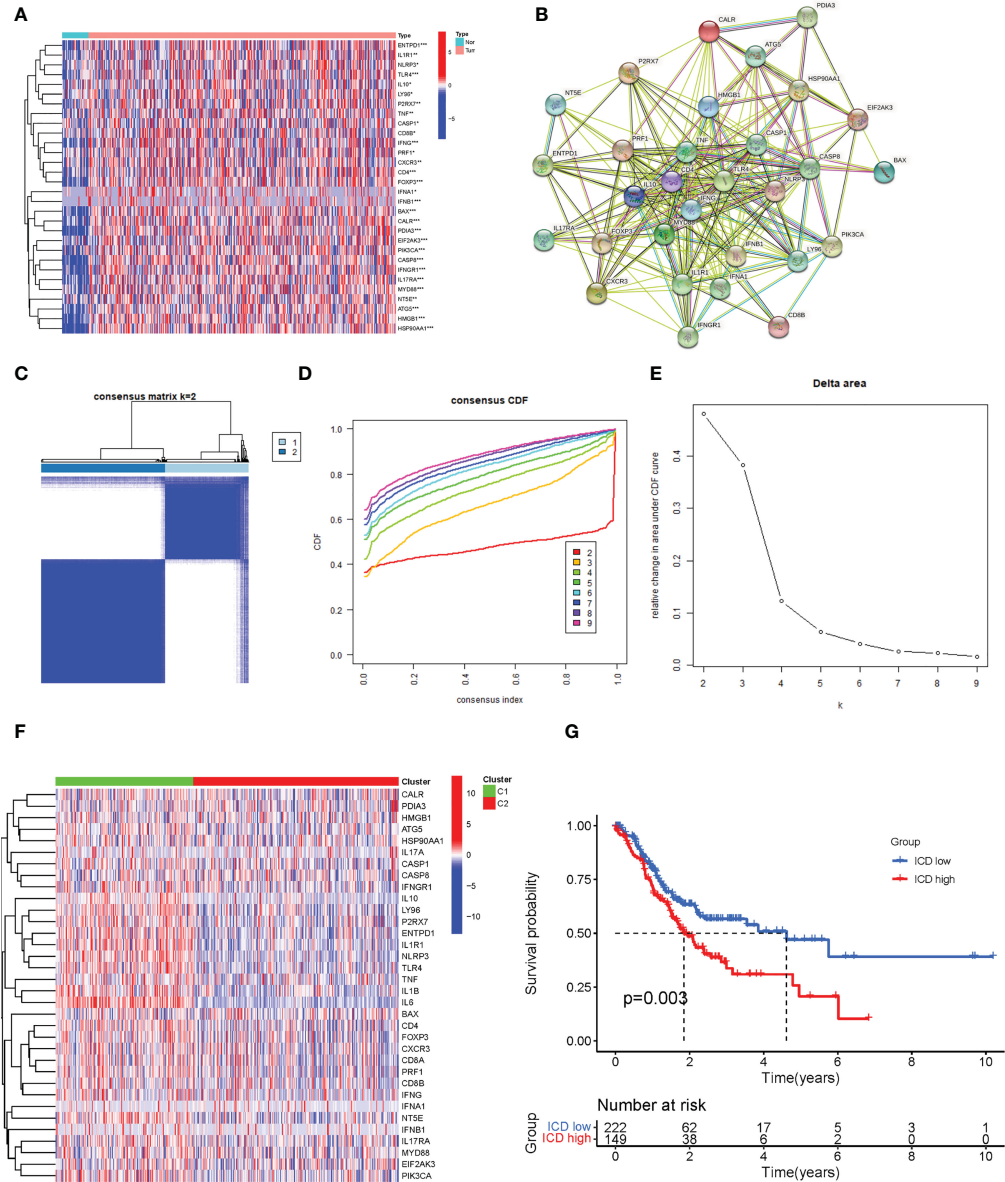
Identification of different immunogenic cell death (ICD)–related subtypes. **(A)** The expression of 34 ICD genes in normal and The Cancer Genome Atlas-Stomach Adenocarcinoma (TCGA-STAD) patients. **(B)** The construction of a protein–protein interaction (PPI) coexpression network of 34 ICD-related genes. **(C)** The heatmap of consensus clustering (k = 2). **(D)** The cumulative distribution function (CDF) curve. **(E)** The relative changes in the area under the CDF curve as the number of clusters varies from k to k + 1. k varies from 2 to 9 and the optimal k = 2. **(F)** The expression of 34 ICD-related genes in different subtypes. **(G)** The Kaplan–Meier (K-M) survival analysis of the two subtypes. *, **, and *** represented p < 0.05, p < 0.01, and p < 0.001, respectively.

The flow chart of the study is presented in **Figure 1**. We identified 34 ICD-related genes from previous studies, including BAX, ATG5, CASP1, CALR, CASP8, CD4, CD8B, CD8A, CXCR3, ENTPD1, EIF2AK3, HMGB1, FOXP3, HSP90AA1, IFNA1, IFNB1, IFNG, IFNGR1, IL10, IL17A, IL17RA, IL1R1, IL1B, IL6, MYD88, LY96, NLRP3, P2RX7, NT5E, PDIA3, PRF1, PIK3CA, TLR4, and TNF. We obtained expression data from 32 normal and 407 STAD tissues in TCGA and further analyzed the expression of these 34 ICD genes, showing that the majority of ICD genes were highly expressed in STAD compared to normal tissues (**Figure 2A**). To further explore the correlations between the ICD genes, a PPI coexpression network was constructed. The two linked genes interact in expression. The constructed PPI coexpression network showed strong interactions between ICD genes (*P*< 0.001) (**Figure 2B**). Based on the ICD gene expression and the survival data of each sample, the samples were clustered using consensus clustering, and we found that the best clusters were the C1 and C2 subtypes when the number of clusters was 2 (**Figures 2C–E**). Then, we calculated the difference in the expression of ICD genes between different subtypes and found that the expression of ICD genes was higher in C1 than in C2 (**Figure 2F**); thus, we defined C1 as the ICD high group and C2 as the ICD low group. Accordingly, the The Cancer Genome Atlas-Stomach Adenocarcinoma (TCGA-STAD) sample was divided into ICD high and ICD low groups. The survival analysis of the two groups showed that the prognosis of the ICD high group was significantly worse than that of the ICD low group (p = 0.003) (**Figure 2G**).

### 3.2 Screening and enrichment analysis of differentially expressed genes based on immunogenic cell death grouping

**Figure 3:**
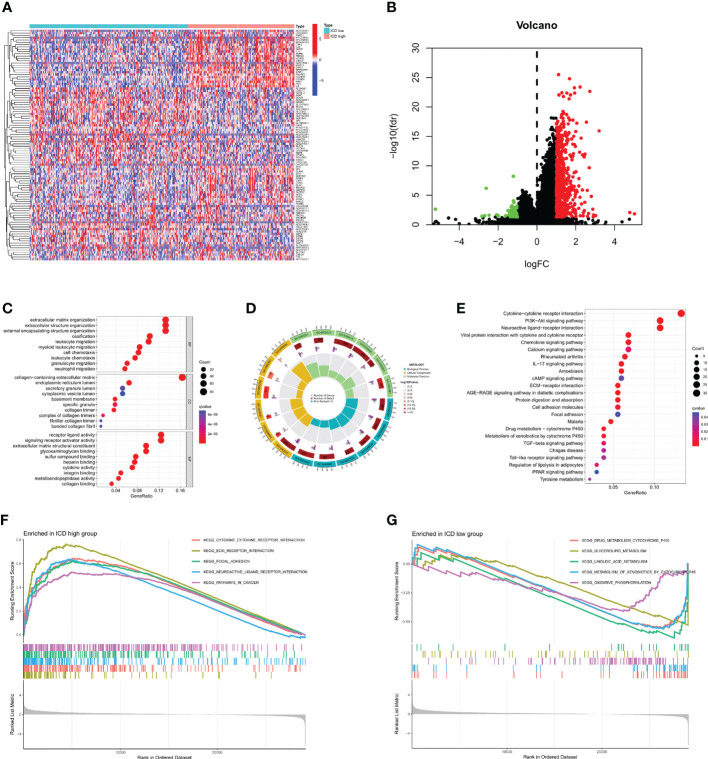
Identification and enrichment analysis of differentially expressed genes (DEGs). **(A)** Identification of DEGs in different subtypes. **(B)** Volcano map of DEGs. **(C, D)** Gene Ontology (GO) enrichment analysis. **(E)** Kyoto Encyclopedia of Genes enrichment analysis. **(F, G)** Gene Set Enrichment Analysis.

After dividing TCGA-STAD into ICD high and ICD low groups, we analyzed the DEGs in both groups (**Figures 3A, B**). A total of 656 DEGs were screened out, and GO enrichment analysis was performed (**Figures 3C, D**). The DEGs were mainly involved in leukocyte chemotaxis, leukocyte migration, and chemokine activity. KEGG enrichment analysis was also performed (**Figure 3E**), and the results showed that the DEGs were mainly involved in cytokine−cytokine receptor interactions, the IL-17 signaling pathway, the chemokine signaling pathway, and the transforming growth factor (TGF)-beta signaling pathway. GSEA was then carried out (**Figures 3F, G**), and the results were similar to those of KEGG. These enrichment analyses all indicated that ICD DEGs were significantly associated with immunity.

### 3.3 Somatic mutation and tumor microenvironment landscape

**Figure 4:**
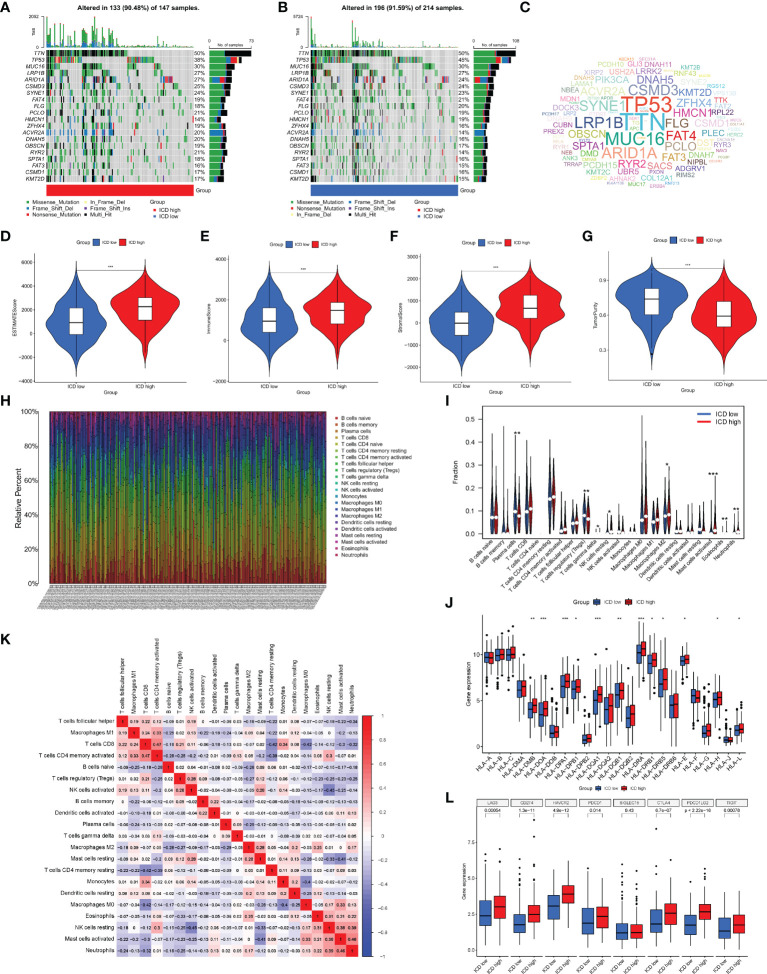
Analysis of somatic mutations and tumor microenvironment (TME) landscape differences between the two subtypes. **(A, B)** Somatic mutations in the ICD-high and ICD-low groups. **(C)** The cloud map of mutant genes based on mutation rates. Violin plots of the ESTIMATE score **(D)**, immune score **(E)**, stromal score **(F)**, and tumor purity **(G)** between the ICD-high and ICD-low groups. **(H)** The proportion of 22 immune cells in each TCGA-STAD cohort. **(I)** The differences in immune cell infiltration between the ICD-high and ICD-low groups. **(J)** Box plots of differentially expressed HLA genes. **(K)** Correlation heatmap of 22 immune cells. **(L)** Box plots of differentially expressed immune checkpoints. *, **, and *** represented p < 0.05, p < 0.01, and p < 0.001, respectively.

Next, we analyzed the somatic mutations, calculated the tumor mutation burden (TMB) for both groups, and plotted the waterfall of the top 20 genes with the highest mutation frequency (**Figures 4A, B**). In the ICD high group, TTN, TP53, MUC16, LRP1B, and ARID1A were the most frequently mutated genes, accounting for 50%, 38%, 30%, 27%, and 27% of the mutations, respectively. In the ICD low group, the genes with the highest mutation frequencies were 50%, 45%, 30%, 27%, and 24%, respectively, as in the ICD high group. The mutant gene cloud was mapped based on mutation rates (**Figure 4C**). Furthermore, among the two groups of mutated genes, the mutation rates of TP53, ARID1A, HMCN1, ZFHX4, DNAH5, and RYR2 were significantly different. It was clear that the gene with the most significant difference in mutation frequency between the two groups was TP53.

Given that enrichment analysis is very relevant to immunity, some analyses were performed on immunity. In the TME, immune cells and stromal cells are the two main types of non-tumor components, and the ratio of immune cells to stromal cells has a significant impact on the tumor prognosis. Thus, we first analyzed the ESTIMATE score (**Figure 4D**), immune score (**Figure 4E**), stromal score (**Figure 4F**), and tumor cell purity (**Figure 4G**). The results showed that the ESTIMATE score, immune score, and stromal score were higher in the ICD high group than in the ICD low group, while the abundance of tumor cells was lower in the ICD high group than in the ICD low group. We next analyzed the differences in the infiltration of 22 immune cells between the two groups, and the ratio of 22 immune cells in GC patients is shown in **Figure 4H**, with the different colors representing the different immune cell types. In addition, violin plots (**Figure 4I**) were used to visualize the differences in immune cell infiltration between the two groups, showing that the degree of infiltration of plasma cells and T-cell regulatory cells (Tregs) was significantly lower in the ICD high group than in the ICD low group, while the infiltration of gamma delta T cells, resting natural killer (NK) cells, M2 macrophages, activated mast cells, eosinophils, and neutrophils showed the opposite trend. The correlation heatmap of 22 immune cells is shown in **Figure 4K**, and most immune cells were negatively correlated with each other. Only a small proportion was positively correlated with each other, for example, activated memory CD4 T cells with CD8 T cells (0.47), neutrophils with activated mast cells (0.46), and activated mast cells with resting NK cells (0.38). The induction of an adaptive antitumor response requires two steps, the most critical of which is the presentation of tumor antigens by the human leukocyte antigen (HLA) to activate CD8 T cells (14). Considering the important role of HLA, we analyzed the expression of each HLA type in the two groups and showed that in the differential HLA-DMB, HLA-DOA, HLA-DPA1, HLA-DPB1, HLA-DQA 1, HLA-DQB1, HLA-DRA, HLA-DRB1, HLA-DRB5, HLA-E, HLA-H, and HLA-L, the ICD high group was significantly higher than the ICD low group (**Figure 4J**). Finally, we analyzed the differences in immune checkpoint expression between the two groups (**Figure 4L**), showing that the expression of almost all immune checkpoints differed between the two groups except for SIGLEC15, and, among the differentially expressed immune checkpoints, the expression in the ICD high group was also significantly higher than that in the ICD low group.

### 3.4 Construction and evaluation of the immunogenic cell death risk signature

**Figure 5:**
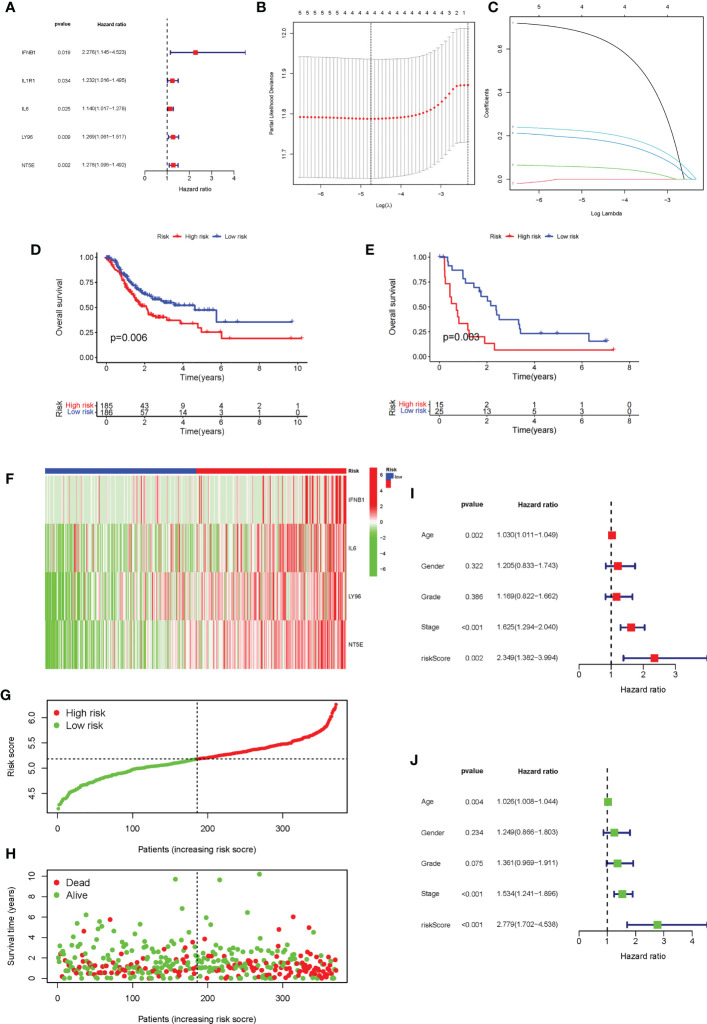
Construction and evaluation of the ICD risk signature. **(A)** Forest plot of univariate Cox analysis for evaluating the prognostic value of the ICD genes. **(B, C)** Lasso Cox analysis identified four ICD-related genes. **(D)** K-M analysis of the risk model in the training dataset TCGA-STAD. **(E)** K-M analysis of the risk model in testing dataset Gene Expression Omnibus data. **(F)** The expression heatmap of the four genes that make up the prognostic signature in the high-risk and low-risk groups of TCGA-STAD. **(G, H)**. Risk score distribution **(F)** and survival status **(G)** of each patient in TCGA-STAD. **(I, J)** Univariate Cox **(H)** and multivariate Cox **(I)** analyses evaluating the association of the risk score and clinical factors with patient OS.

The univariate Cox analysis of ICD-related genes was performed, and five prognosis-related genes were identified: IFNB1 (*P* = 0.019), IL1R1 (*P* = 0.034), IL6 (*P* = 0.025), LY96 (*P* = 0.009), and NT5E (p = 0.02), followed by forest plotting (**Figure 5A**). We use the TCGA-STAD as the training dataset and GEO28541 as the testing dataset. Based on these five genes, LASSO regression analysis was performed, from which four genes, IFNB1, IL6, LY96, and NT5E, were screened for the construction of the risk signature (**Figures 5B, C**), and the coefficients of the four genes are presented in **Supplementary Table**. We verified this signature from many aspects. First, according to the prognostic signature, the TCGA and GEO data were divided into high-risk and low-risk groups, and then survival analysis was carried out. The results showed that patients in the low-risk group had a better survival prognosis (P = 0.006 and P = 0.003) (**Figures 5D, E**). We analyzed the expression of the four genes that make up the prognostic signature in the two groups of TCGA-STAD samples and found that these four genes showed high expression in most samples in the high-risk group and low expression in the low-risk group (**Figure 5F**). Furthermore, we analyzed the survival status of patients in TCGA-STAD, and patients in a state of death tended to have high-risk scores (**Figures 5G, H**). Finally, we performed univariate and multifactorial Cox analyses, showing that ICD-related risk scores were an independent prognostic predictor of OS in patients with GC (**Figures 5I, J**).

### 3.5 Correlation of risk scores with immune cells and predictive role for immunotherapy

**Figure 6:**
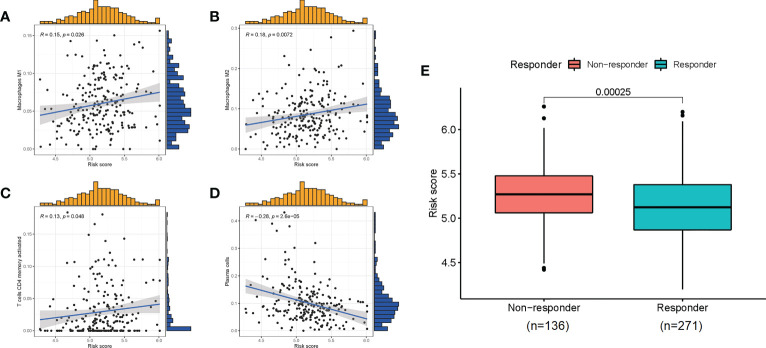
The correlation of risk scores with immune cells **(A–D)** and the predictive role for immunotherapy **(E)**.

**Figure 7:**
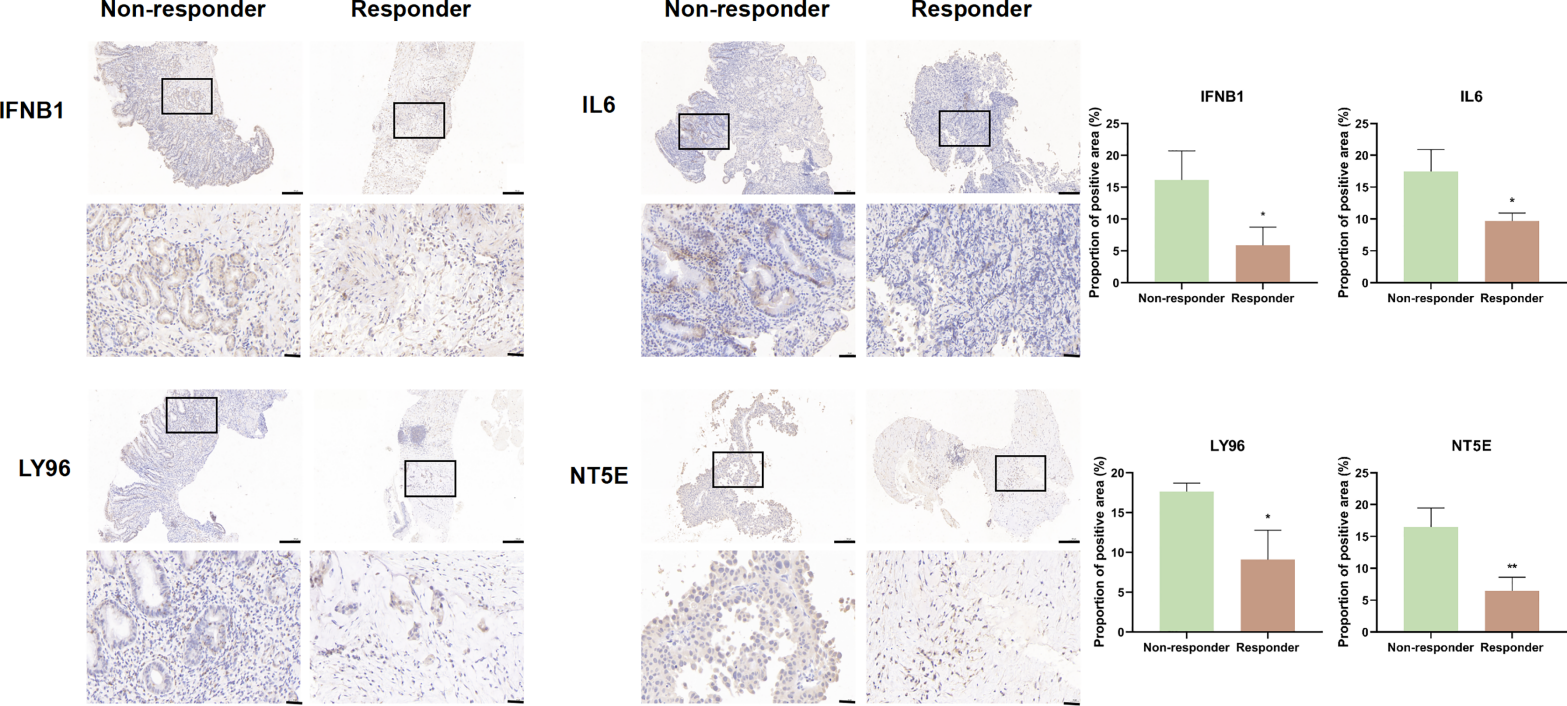
IFNB1, IL6, LY96, and NT5E expression was verified by IHC in clinical tissues that responded or non-responded to immunotherapy (scale bar: 200 and 40 μm).

Given the role of ICD-related genes in the immune microenvironment, we analyzed the correlation between risk scores and immune cells. We found that risk scores showed a positive correlation with the number of M1 macrophages (**Figure 6A**), M2 macrophages (**Figure 6B**), and activated memory CD4 T cells (**Figure 6C**) and a negative correlation with plasma cells (**Figure 6D**). Furthermore, the impact of risk scores on ICI therapy was assessed using Tumor Immunity Functioning (TIDE) (http://tide.dfci.harvard.edu/query/), and it was found that the risk score for non-responders was significantly higher than that for responders, suggesting that the ICD risk score was a good predictor of immunotherapy effectiveness (**Figure 6E**). We used clinical tissues to validate the predictive effect of this signature on immunotherapy. Then, immunohistochemistry (IHC) was performed to investigate the differences expression of the four genes that make up the signature between patients who responded or did not respond to immunotherapy. Encouragingly, we found a lower expression of these four genes in patients who responded to immunotherapy compared to non-responders, which is consistent with our study where patients with high-risk scores responded less well to immunotherapy (**Figure 7**).

### 3.6 Assessing protein expression of genes comprising the immunogenic cell death risk signature using immunohistochemistry

**Figure 8:**
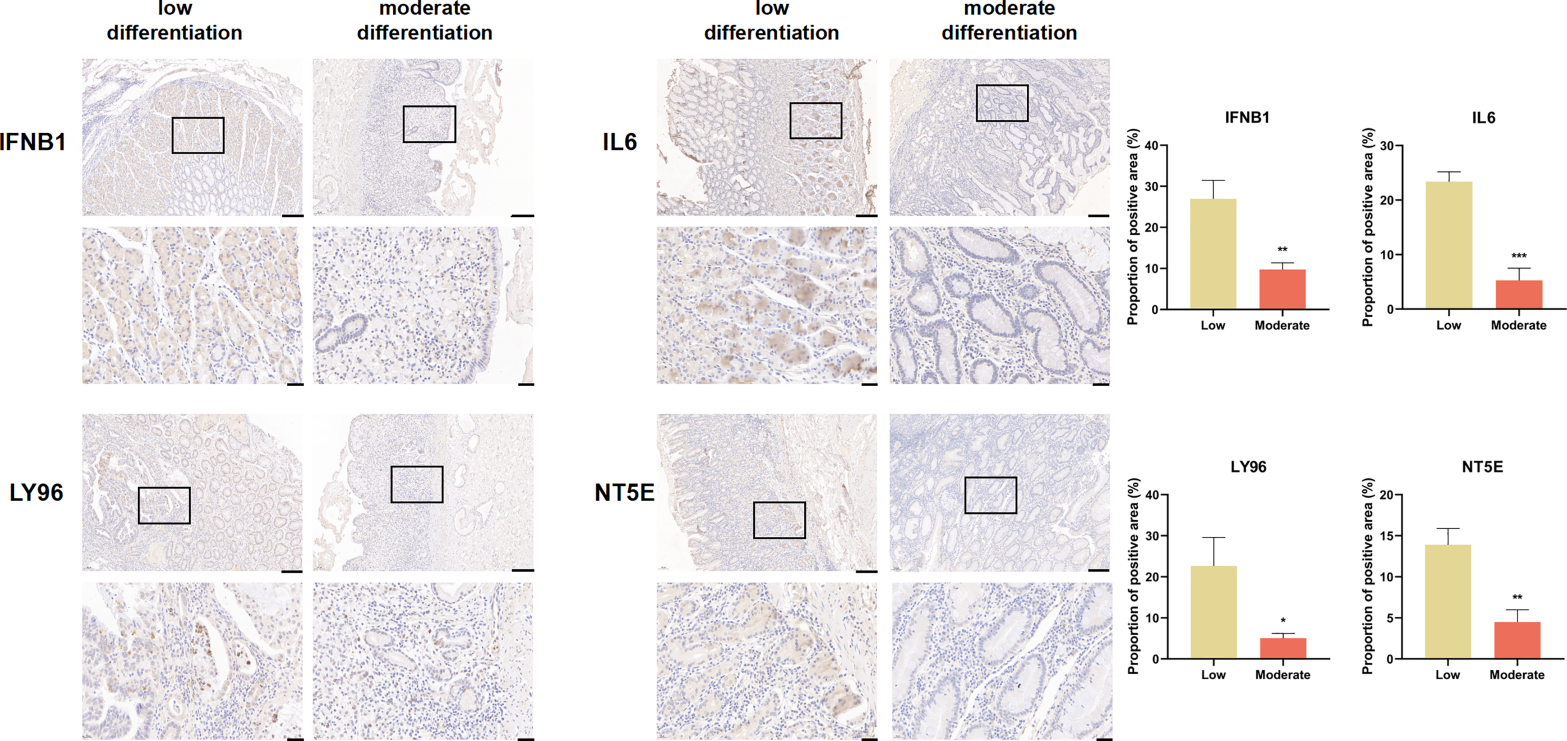
IFNB1, IL6, LY96, and NT5E expression were verified by IHC in clinical tissues (scale bar: 200 and 40 μm).

We explored the expression of genes comprising the ICD risk model, including IFNB1, IL6, LY96, and NT5E, by IHC using pathological sections from GC patients of different pathological grades. The results showed that the expression of these four genes was significantly higher in patients with high malignancy than in those with low malignancy (**Figure 8**).

## 4 Discussion

Over the past few years, comprehensive treatments for GC have developed rapidly, especially in the area of immunotherapy, which has made significant progress. ICI therapy is an emerging cancer treatment that targets immune checkpoint proteins on the surface of immune cells or tumor cells to activate antitumor immunity for tumor-killing effects (15). Several clinical trials have shown that ICI therapy has controlled toxicity and good antitumor effects in GC patients (16). However, different studies have shown that the objective remission rate of ICI therapy in GC varies widely from 10% to 26% (17). Not all patients produce a good response. It is therefore particularly important to increase the proportion of patients who benefit from immunotherapy and the effectiveness of immunotherapy. There have been many studies focusing on immunotherapeutic biomarkers for GC (18–20), such as PD1/programmed cell death 1 ligand 1 (PD-L1) immunohistochemical staining, TMB, and MSI, but all of them have some problems: programmed cell death 1 ligand 1 (PD-L1) IHC results are not fully consistent with the patient response; there is no accepted standard formula for the calculation of TMB, nor is there an absolutely definitive threshold to distinguish between high and low tumor mutation load in patients; the evaluation of MSI is influenced by the spatial heterogeneity within the tumor; and different results may be obtained when different parts of the tumor are evaluated (21). Therefore, the search for the development of new biomarkers holds great promise in screening sensitive patients for precise therapies for tumor ICIs. Therefore, the search for new biomarkers is of great importance in screening sensitive patients for precise treatment with tumor ICI therapy.

In this study, we analyzed the expression of 34 ICD genes and identified two subtypes based on consensus clustering analysis: ICD high and ICD low groups. Patients in the ICD-high subtype tended to have longer OS than those in the ICD low group. Moreover, the two subtypes also differed significantly in genetic mutation, tumor cell purity, HLA gene expression, and immune checkpoint expression. Precision medicine in the era of tumor immunotherapy may be supported by multiparametric biomarkers; therefore, we built an immune-related signature to predict the prognosis of GC patients based on four hub genes and found that this predictive model could well predict the outcomes of immunotherapy.

Somatic mutations are considered critical in disease, and different mutational states may influence tumor progression, clinical outcomes, and treatment strategies (22). Somatic mutations help determine the degree of tumor heterogeneity, and TMB is an indicator of the total number of somatic mutations in a particular tumor. Some of these mutations encode neoantigens, which are presented and promote the rejection of the tumor by T cells. Thus, the more mutations there are in the tumor, the more neoantigens may be present (23). In this study, we analyzed the somatic mutations in the ICD high and ICD low groups and measured the TMB for each group. Among the two groups of mutated genes, the mutation rates of TP53, ARID1A, HMCN1, ZFHX4, DNAH5, and RYR2 were significantly different. Some studies have shown that TP53 is one of the most widely studied oncogenes, and its mutation not only eliminates the tumor suppressor function but also produces certain pro-cancer functions (24). ARID1A deletion induces the chemotaxis of polymorphonuclear bone marrow–derived suppressor cells, the main type of invasive immune cell that causes immune evasion, and promotes the progression of prostate cancer (25). In addition, ARID1A mutations are associated with extensive DNA damage repair defects and an immunogenic TME (26). HMCN1 belongs to the extracellular matrix (ECM) protein family and encodes an extracellular protein of the immunoglobulin superfamily. HMCN1 is mutated and aberrantly expressed in a variety of tumors, and, in ccRCC, HMCN1 mutations are a key event in tumor progression (27). In breast cancer, intratumoral heterogeneity of HMCN1 mutant alleles is associated with a poor patient prognosis (28), and mutations in HMCN1 occur in approximately 60% of patients, regardless of the MSI status in GC patients (29). There are frequent mutations in ZFHX4 in esophageal squamous cell carcinoma (30). Meanwhile, DNAH5 has a high mutation rate in myeloma (31). RYR2 mutation enhances the antitumor immune response by activating memory M1 macrophages and CD4+ T cells as well as enriching CD8 T cells (32). Therefore, mutations in these genes significantly influence the development of tumors. Consistent with the literature, mutations in these genes also affect the progression of stomach cancer.

We developed a prognostic signature based on ICD-related genes, including IFNB1, IL6, LY96, and NT5E. The model was validated in several ways, including survival analysis, the expression of hub genes, the correlation between the risk score and the patient survival status, and univariate and multifactor Cox analyses, showing that the risk score was an independent prognostic factor for predicting OS, showing good predictive accuracy in STAD patients. Current research has found that the TME is closely related to tumorigenesis, development, and the prognosis and that immune cells exhibit complex interactions with tumor cells. Therefore, the correlation between the risk score and immune cells was analyzed. Risk scores showed a positive correlation with activated memory CD4 T cells, M1 macrophages, and M2 macrophages and a negative correlation with plasma cells. Macrophages are classified into two subgroups, M1 macrophages and M2 macrophages, based on their function and the level of inflammatory factor secretion. M1 macrophages (classically macrophages), mainly activated by Interferon γ (IFNγ); lipopolysaccharide (LPS), secrete low levels of IL10 and high levels of IL2, mainly to promote the development of inflammation and phagocytosis (33). M2 macrophages (alternatively macrophages) are activated by IL4 inflammatory factors and play a role in processes such as wound healing and tissue repair, mainly through the secretion of anti-inflammatory cytokines such as IL10 (34). The dysregulation of the M1/M2 ratio in the TME plays a key role in disease processes such as tumor development, immune escape, and drug resistance (35), and therefore, a stable M1/M2 ratio is essential for the TME. CD4 memory T cells are an important component of the adaptive immune response. ICI-related immunotherapy has received much attention as the most promising treatment modality available (36). We therefore examined the impact of risk scores on ICI therapy, and ICD risk scores can be a good predictor of the effectiveness of immunotherapy.

Our research still has certain limitations. First, the clinical data we downloaded from the public database are incomplete and lack some important clinical details, and this study lacks the validation of animal models for predicting the effects of immunotherapy. We need to conduct a prospective and multicenter large-sample study to verify the accuracy of the ICD signature we established.

## 5 Conclusions

This study established and validated an ICD-related signature to accurately predict OS and immunotherapy response in GC patients and showed potent predictive power. The above results may help to deepen our understanding of ICD and provide new strategies for personalized treatment.

## Data availability statement

The original contributions presented in the study are included in the article/**Supplementary Material**. Further inquiries can be directed to the corresponding authors.

## Ethics statement

The studies involving human participants were reviewed and approved by Biomedical Research Ethics Committee of Peking University First Hospital. Written informed consent for participation was not required for this study in accordance with the national legislation and the institutional requirements.

## Author contributions

XP, XW, and YC conceived, designed, and supervised the overall study; BD, YW, and JZ performed the statistical analyses, prepared the figures and tables, and wrote the manuscript; YG, RX and XH performed Immunohistochemistry. All authors have read and approved the article.
