# Supplementary material for: A novel immunogenic cell death–related subtype classification and risk signature for predicting prognosis and immunotherapy efficacy in gastric cancer
Source: Front Immunol. 2023 May 5;14:1162876. doi: 10.3389/fimmu.2023.1162876 (PMC10196197; doi:10.3389/fimmu.2023.1162876)
Supplement: Supplementary file 1 [file Table_1.doc]

Gene Coef

IFNB1 0.827389144386875

IL6 0.0447649287590016

LY96 0.162832850954981

NT5E 0.179025394290104
